# Immune recovery in HIV-1 infected patients with sustained viral suppression under long-term antiretroviral therapy in Ethiopia

**DOI:** 10.1371/journal.pone.0240880

**Published:** 2020-10-22

**Authors:** Dawit Wolday, Dorsisa Legesse, Yazezew Kebede, Dawd S. Siraj, Joseph A. McBride, Robert Striker

**Affiliations:** 1 Mekelle University College of Health Sciences, Mekelle, Ethiopia; 2 Hayat General Hospital, Addis Ababa, Ethiopia; 3 Division of Infectious Diseases, Department of Medicine, University of Wisconsin, Madison, Wisconsin, United States of America; Instituto de Salud Carlos III, SPAIN

## Abstract

**Background:**

There is very little data on long-term immune recovery responses in patients on suppressive antiretroviral therapy (ART) in the setting of sub-Saharan Africa (SSA). Thus, we sought to determine CD4+ T-cell, CD8+ T-cell and CD4/CD8 ratio responses in a cohort of HIV infected individuals on sustained suppressive ART followed up for more than a decade.

**Methods:**

The cohort comprised adult patients who started ART between 2001 and 2007 and followed for up to 14 years. Trends in median CD4+ T-cells, CD8+ T-cells and CD4/CD8 ratio were reviewed retrospectively. Poisson regression models were used to identify factors associated with achieving normalized T-cell biomarkers. Kaplan-Meier curves were used to estimate the probability of attaining normalized counts while on suppressive ART.

**Results:**

A total of 227 patients with a median duration of follow-up on ART of 12 (IQR: 10.5–13.0) years were included. CD4 cell count increased from baseline median of 138 cells (IQR: 70–202) to 555 cells (IQR: 417–830). CD4 cell increased continuously up until 5 years, after which it plateaued up until 14 years of follow up. Only 69.6% normalized their CD4 cell count within a median of 6.5 (IQR: 3.0–10.5) years. In addition, only 15.9% of the cohort were able to achieve the median reference CD4+ T-cell threshold count in Ethiopians (≈760 cells/μL). CD8+ T-cell counts increased initially until year 1, after which continuous decrease was ascertained. CD4/CD8 ratio trend revealed continuous increase throughout the course of ART, and increased from a median baseline of 0.14 (IQR: 0.09–0.22) to a median of 0.70 (IQR: 0.42–0.95). However, only 12.3% normalized their ratio (≥ 1.0) after a median of 11.5 years. In addition, only 8.8% of the cohort were able to achieve the median reference ratio of healthy Ethiopians.

**Conclusion:**

Determination of both CD4+ and CD8+ T-cells, along with CD4/CD8 ratio is highly relevant in long-term follow-up of patients to assess immune recovery. Monitoring ratio levels may serve as a better biomarker risk for disease progression among patients on long-term ART. In addition, the findings emphasize the relevance of initiation of ART at the early stage of HIV-1 infection.

## Introduction

Effective antiretroviral therapy (ART) suppresses HIV replication allowing progressive immune recovery which is used commonly to assess the status of disease progression [1, 2]. ART programs in sub-Saharan Africa (SSA) have achieved outcomes comparable to those in developed countries, especially in terms of immunological and virological responses despite differences in HIV-1 genetic diversity [3–6].

Reports on outcomes to ART in patients followed up for more than a decade are documented mostly from high-income countries (HICs) [7–12]. On the other hand, reports from SSA on outcomes of ART are those of short duration and long-term treatment outcomes, by large, are hardly known despite the fact that patients are living longer [13, 14]. In addition, there are no data with respect to long-term CD4/CD8 ratio responses among patients taking ART in SSA. Despite effective viral suppression, however, immune recovery is often incomplete, which very often remains below the normal threshold levels [15–24]. Incomplete immune recovery despite ART has been linked with increased mortality and morbidity, both due to AIDS-related and non-AIDS-related or non-communicable diseases [24–34], and monitoring the ratio appears to be both relatively simple and comprehensive way of evaluating changing risks for clinical events.

We have previously shown that apparently healthy HIV-negative adult Ethiopians exhibit low CD4+ T-cell count and CD4/CD8 ratio compared to Caucasians [35–39]. Despite low baseline CD4+ T-cell count or CD4/CD8 ratio among Ethiopians, HIV disease progression during pre-ART era remained the same in the two populations [38]. In settings, like ours, very low CD4+ T-cell count and low CD4/CD8 ratio in apparently healthy HIV-seronegative individuals poses challenge for ART guidelines based on normal reference threshold values. Hence, we sought to determine immune recovery among Ethiopian patients initiated with ART followed up for more than a decade. In addition, we determined factors associated with immune recovery in this patient population.

## Methods

### Study population

The cohort was enrolled at Hayat General Hospital’s ART Clinic in Addis Ababa, Ethiopia, which delivers HIV treatment and care [40]. The cohort consists of adult patients followed between 2001 and 2017. Patients with an AIDS defining illness or a CD4 count <200 cell/μL were started on with a combination of ART, based on National ART Guidelines [41]. First-line ART comprised of zidovudine (AZT) or stavudine (d4T) or tenofovir (TDF) in combination with lamivudine (3TC) plus non-nucleoside reverse transcriptase inhibitors, nevirapine (NVP) or efavirenz (EFV). The participants had clinic visits every 6 months and/or as needed for monitoring treatment outcomes. Laboratory tests performed during each visit included complete blood cell count, clinical chemistry, urine analysis, and immune-profiles (as measured by CD4 cell count, CD8 cell count and CD4/CD8 ratio). CD4, CD8 and CD4/CD8 ratio was measured using FACSCount (BD Biosciences, San Jose, USA). HIV-1 plasma viral load determination was done by real-time nucleic-acid sequence-based amplification (NASBA) using the NUCLISENSE EASYQ technology (BioMérieux, Boxtel, The Netherlands). The lower limit of detection (LDL) of the assay is 50 RNA copies/mL blood. HIV-1 viral load was done for patients in the event of clinical or immunological failure, as per National Guidelines [41]. Clinical failure is suspected when new or recurring WHO clinical stage III or IV event occurs, and immunological failure is considered when the CD4 count is persistently < 250 cells/μL, or there is persistent CD4 count levels < 100 cells/μL.

Patients were censored at the time of CD4, CD8 or CD4/CD8 ratio normalization, at the time of death, transfer to other clinic, loss to follow-up, or the last visit date before December 2017. CD4 count, CD8 count and CD4/CD8 ratio are considered normalized if ≥ 500 cells, between 500 and 1000 cells, and ≥ 1.0, respectively (based on values of healthy adult Ethiopians [35–39]). Viral suppression was defined as the attainment of HIV-1 plasma viral load of <400 copies/mL on at least two subsequent measurements. Poor adherence was defined as intake of prescribed medications <95%, based on National ART Guidelines [41]. Adherence was monitored based on pill counts during clinic visits, self-report as well as by checking pharmacy refill records.

### Statistical analysis

Baseline characteristics for continuous variables were expressed as the median with interquartile range (IQR), and for categorical variables as proportions. Chi-square test was used to compare categorical variables and Wilcoxon rank-sum test for not normally distributed variables.

The rate of T-cell marker normalization was calculated per 100 person years at risk. Factors associated with probability of T-cell marker normalization were analyzed using Poisson regression analysis. Using univariate analysis, we initially analyzed the relative risk (RR) of not achieving CD4+ T-cell count normalization with respect to baseline demographic and clinical characteristics (age, gender and HIV disease stage, TDF-based regimen), presence of tuberculosis (TB) at time of ART initiation and baseline laboratory biomarkers including CD4+ T-cell count, CD8+ T-cell count, CD4/CD8 ratio and plasma viral load. In addition, time-dependent variables including drug toxicity, ART adherence, ART switching, as well as updated T-cell markers were included in the initial bivariate analysis. Then a multivariate analysis of probability [adjusted relative risk (ARR)] of achieving CD4 count ≥ 500 cells was done including all variables that were significant in univariate analysis.

Baseline CD4+ T-cell counts were built in to two categories whether normalized (≥500) or not normalized (<500) cells/μL, the CD8 cell counts in three categories (<500, between 500 and 1000, and >1000 cells/μL), and the CD4/CD8 ratio in two categories (<0.30 and ≥0.30). The cut-offs were chosen based on previous reports showing association of the biomarkers with AIDS-related events [31, 32, 40].

Kaplan-Meier curves were used to estimate the probability of CD4+ T-cell count normalization in patients on ART across explanatory variables and statistical differences were tested using log-rank test. P values <0.05 were considered statistically significant. Data was entered on excel and exported for analysis by STATA (Statistical package v. 14.0, StataCorp, Texas, USA).

### Ethical considerations

The study was reviewed and approved by the Addis Ababa City Administration Health Bureau Research Ethics Review Committee, Addis Ababa, Ethiopia and the Institutional Review Board (IRB) of the University of Wisconsin, Madison, USA. As the nature of the study was retrospective, individual study participant consent was not obtained and approved by the ethics review committee. Nonetheless, all personal identifiers were de-linked from the original sources.

## Results

### Characteristics of study participants

Socio-demographic and baseline clinical features are shown in Table 1. A total of 227 patients receiving ART and with sustained virological suppression were included in the study. The median age at ART initiation was 39 years (IQR: 33–46) years. The majority were male (63.4%) with more than half (56.8%) being in Stage III and IV WHO clinical stage.

**Table 1:** Sociodemographic and clinical characteristics.

Variable	Overall
**Total patients**	227 (100.0)
**Age**	
Median years, (IQR)	39 (33–46)
**Sex**	
Male	144 (63.4)
Female	83 (36.6)
**WHO Clinical Stage**	
I/II	98 (43.2)
III/IV	129 (56.8)
**Adverse drug effects**	
No	203 (89.4)
Yes	24 (10.6)
**ART adherence**	
Good	210 (92.5)
Poor	17 (7.5)
**ART switching**	
No	86 (37.9)
Yes	141 (62.1)
**CD4 cell count (cells/μL) at baseline**	
Median (IQR)	138 (70–202)
< 200	167 (74.6)
≥ 200	57 (25.4)
**CD8 cell count (cells/μL) at baseline**	
Median (IQR)	899 (645–1266)
< 500	25 (12.1)
500–1000	93 (45.2)
>1000	88 (42.7)
**CD4/CD8 ratio at baseline**	
Median (IQR)	0.14 (0.09–0.22)
< 0.30	178 (86.4)
≥ 0.30	28 (13.6)
**Viral load (RNA copies/mL) at baseline**	
Median (IQR)	120 000 (27 000–280 000)
**Follow-up [median (IQR) Years]**	12.0 (10.5–13.0)
**ART regimen**	
AZT+3TC+ EFV	91 (40.1)
d4T+3TC+ EFV	60 (26.4)
d4T+3TC+ NVP	31 (13.7)
TDF+3TC+EFV	18 (7.9)
AZT+3TC+ NVP	16 (7.1)
TDF+3TC+NVP	4 (1.8)
PI containing	6 (2.6)
Other	2 (0.9)

Data are numbers (%) unless otherwise stated. ART = antiretroviral therapy; IQR = interquartile range.

### Trends of T-cell biomarkers

**Fig 1:**
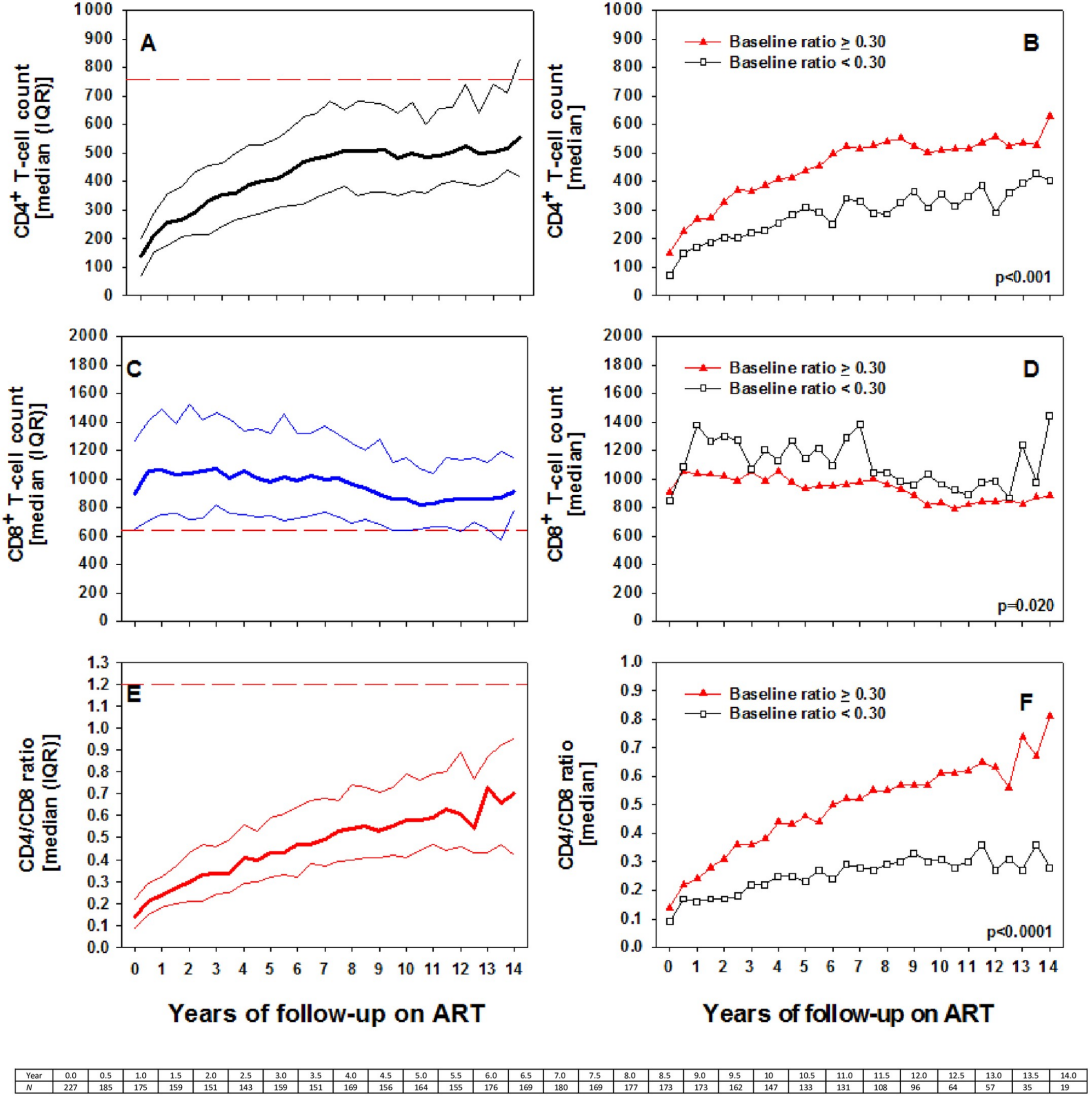
Trends in CD4+ T-cell (A), CD8+ T-cell (C) counts and CD4/CD8 ratio (E) among all patients with virologically suppressive ART, or trends in CD4+ T-cell (B), CD8+ T-cell (D) counts and CD4/CD8 ratio (F) in those stratified by baseline ratio < 0.30 or ≥ 0.30. Data represent median (IQR) for A, C and E, or as median only for B, D and F. Dashed horizontal lines across figures A, C and E represent normal reference threshold values in healthy Ethiopians [34].

Trends of the CD4+ T-cell, CD8+ T-cell and CD4/CD8 ratio is shown in Fig 1. The CD4 cell count increased from baseline median of 138 (IQR: 70–202) to 555 (IQR: 417–830) cells/μL after a median of 12 (IQR: 10.5–13.0) years of follow up on ART (p<0.0001) (Fig 1A). The increase of CD4 T cells exhibited a biphasic pattern. It increased continuously from baseline median of 138 (IQR: 70–202) to 510 (IQR: 385–653) cells/ μL after a median of 7.5 years of follow up on ART (p<0.00001). However, beyond 7.5 years of ART, the CD4+ T-cell count plateaued and there is no significant change when the median CD4 cell count at year 5 was compared to the median CD4 cell count at year 14 of follow up on ART (p = 0.8588). Whereas the median increase of CD4 count per year (slope) was 38 (IQR: 30–57) cells/μL in phase one, it plateaued at around -1 (-13–25) cells/μL per year in phase two of the follow-up (p = 0.0109). Continuous increases in the median CD4+ T-cell counts in both groups with baseline CD4/CD8 ratio of less than 0.30 and ≥ 0.30 were observed, though increases were more significant in the group with baseline CD4/CD8 ratio of ≥ 0.30 (Fig 1B). The median increase of CD4 count per year in those with baseline CD4/CD8 ratio ≥ 0.30 was 43 (IQR: 28–53) cells/μL up until 8.5 years compared to only 7 (IQR: -18–33) cells/μL after 8.5 years of follow-up. In addition, the median increase of CD4 count per year in those with baseline ratio < 0.30 was 33 (IQR: 16–55) cells/μL up until 5 years, but only 4 (IQR: -18–10) cells/μL after 5 years of follow-up. Overall, only 69.6% of the cohort has normalized CD4 cell count (≥500 cells) during the 2631.6 person-years of follow-up, and the median time to CD4 count normalization was 6.5 (IQR: 3.0–10.5) years. In addition, only 15.9% of the cohort were able to achieve the median reference CD4+ T-cell threshold count in Ethiopians, which is ≈760 cells [35]. The median time to reach the reference CD4 cell count threshold of Ethiopians was 12 (IQR: 10.8–13.3) years.

Trends in CD8 T cell count exhibited a three phase pattern, with initial increase up until 1 year, followed by plateau until 6 years and decline after 6 years of ART follow-up (Fig 1C). CD8+ T-cell counts increased initially until year 1, after which continuous decrease in CD8+ T-cell count was ascertained. Overall, there was no significant change in CD8+ T-cells when baseline counts were compared to those at 14 years. However, there was statistically significant decrease in CD8+ T-cells when median count at year 1 [1063 (IQR: 754–1490) cells/μL was compared to the median count at year 14 [911 (IQR: 788–1154) cells/μL] on ART (p = 0.005). Although decrease in CD8+ T-cell count between year 1 and 6 was not significant, significant decrease was noted between year 6 and year 14 of follow up [986 (IQR: 730–1322 cells/μL) vs. 911 (IQR: 788–1154 cells/μL), p = 0.009]. In the early phase until year 1, the median increase of CD8 count per year was 81(IQR: 0–162) cells/μL. The median change of CD8 count per year between the years 1 and 6 was -28 (IQR: -35–1) cells/μL, and between the years 7 and 14 was 3 (IQR: -41–10) cells/μL. However, the changes were not statistically significant. Although, the median CD8 count decline per year was -23 (-41–10) cells/μL after 1 year of follow-up, it was not statistically significant compared to the slope noted up until 1 year (p = 0.2341). CD8 T cell trends stratified by CD4/CD8 ratio did not show any pattern (Fig 1D).

The CD4/CD8 ratio trends revealed continuous increase throughout the course of ART, and increased from a median baseline of 0.14 (IQR: 0.09–0.22) to a median of 0.70 (IQR: 0.42–0.95) at the time of censoring the study (p = 0.0003) (Fig 1E). However, only 12.3% of the cohort has normalized ratio (≥ 1.0). The median time to ratio normalization was 11.5 (95% CI: 10.0–12.5) years. Continuous increases in the median CD4/CD8 ratio were more significant in the group with a baseline ratio of ≥ 0.30 than in those with less than 0.30. In the latter group, slight increase in ratio were noted up until year 4 on ART, then it plateaued (Fig 1F). The overall CD4/CD8 ratio increase per year (slope) was 0.04 (IQR: 0.02–0.05). The median increase of CD4/CD8 ratio per year in those with baseline ratio ≥ 0.30 was 0.03 (IQR: 0.02–0.06). On the other hand, the trend in ratio change for those with a baseline < 0.30 showed somewhat biphasic pattern. Whereas the median increase in CD4/CD8 ratio up until 9 years of follow-up was 0.02 (IQR: 0.01–0.04), it decreased by a median ratio of -0.01 (IQR: -0.02–0.03) beyond 9 years. However, the difference was not statistically significant (p = 0.0847). In addition, only 8.8% of the cohort were able to achieve the median reference ratio threshold value of healthy Ethiopians, which is ≈1.10 [35]. Overall, the proportion of patients with a CD4/CD8 ratio of ≥ 0.3, ≥ 0.5, ≥0.8, ≥ 1.0 and ≥ 1.2 were 85.5%, 57.3%, 26.9%, 12.3% and 6.6%, respectively (S1 Fig).

### Factors associated with immune recovery

Predictors associated with failure to achieve CD4+ T-cell normalization ≥ 500 is shown in Table 2. On univariate analysis female sex (RR: 2.26, 95% CI: 1.26–4.07), CD4+ T-cell count < 200 cells at baseline (RR: 2.93, 95%CI 1.34–6.24), and CD4/CD8 ratio at baseline (per 0.1 point lower) (RR: 1.57, 95%CI 1.19–2.07) were all associated with failure to achieve CD4+ T-cell normalization. On the contrary, age, WHO clinical stage, adverse drug events, adherence, ART switching, TDF-based regimen, prevalent or incident TB, AIDS-defining events, non-AIDS-defining events, CD8+ T-cell count and baseline plasma viral load were all not associated with failure to achieve CD4 normalization.

**Table 2:** Factors associated with not achieving CD4+ T-cell count ≥ 500 in HIV-infected patients on antiretroviral treatment despite sustained viral suppression.

Variable	Univariate	Multivariate		
	RR (95% CI)	*P*-value	ARR (95% CI)	*P*-value
**Age (years)** ≥ 50 vs. < 50	1.75 (0.90–3.43)	0.100		
**Gender, male vs female**	2.26 (1.26–4.07)	0.006	1.64 (0.88–3.06)	0.120
**WHO stage** III/IV vs. I/II	1.11 (0.69–1.80)	0.664		
**Adverse drug events vs none**	1.11 (0.53–2.32)	0.783		
**Poor vs good adherence**	0.97 (0.39–2.40)	0.939		
**ART switch vs. no switch**	0.95 (0.59–1.54)	0.831		
**TB at ART initiation vs. no TB**	1.86 (0.85–4.06)	0.120		
**Incident TB vs. no TB**	1.53 (0.80–2.92)	0.194		
**AIDS-defining events vs. none**	1.29 (0.74–2.22)	0.367		
**Non-AIDS-defining events vs none**	1.29 (0.74–2.26)	0.369		
**CD4+ T-cell count at baseline**< 200 vs. ≥ 200	2.93 (1.34–6.40)	0.007	1.82 (0.78–4.21)	0.165
**CD8+ T-cell count at baseline**				
500–1000	1	--		
500	0.55 (0.21–1.40)	0.208		
>1000	0.78 (0.46–1.30)	0.338		
**CD4/CD8 ratio at baseline** (per 0.1 lower)	1.57(1.19–2.06)	0.002	1.36 (1.01–1.82)	0.041
**Viral load at baseline (RNA copies/mL)** ≥ 100,000 vs. < 100,000*	0.98 (0.41–2.33)	0.964		

RR: relative risk; ARR: adjusted relative risk;

*Data available for only 59 patients at baseline.

In multivariate analysis, however, baseline CD4/CD8 ratio (per 0.1 point lower) (ARR: 1.36, 95% CI: 1.01–1.82, p = 041) was the only factor associated with increased probability of failure to achieve CD4+ T-cell normalization.

## Discussion

Data on long-term outcomes of ART in the setting of SSA are limited [13, 14]. Here, we measured CD4+ T-cell count, CD8+ T-cell count and CD4/CD8 ratio during a median of 12 years with sustained virological suppression in HIV-1 infected patients on ART.

The CD4+ T-cell trends showed continuous increase up until year 5–7 of follow up, but plateaued thereafter. Median reference CD4+ T-cell count [35] was achieved within an average of 12 years follow-up in only 15% of the patients. In addition, using multivariate analysis, the likelihood of achieving CD4+ T-cell count normalization (≥ 500 cells) was significantly associated with time-updated CD4/CD8 ratio ≥ 0.30 at ART initiation. In addition, similar to previous studies undertaken in HICs [7–12] and SSA [13, 14], our findings showed that the majority of the patients (76%) started ART with a CD4+ T-cell lower than 200 cells. Only 4 patients were initiated ART with their CD4+ T-cell counts ≥ 350 cells. Therefore, we did not analyze the effect on CD4+ T-cell normalization when patients were initiated with ART when their CD4+ T-cell count was ≥ 350 cells. Nonetheless, several studies conducted previously have demonstrated that baseline CD4+ T-cell count ≥ 350 is a significant predictor of achieving CD4+ T-cell normalization [9, 12, 23, 24]. In our study, baseline CD4/CD8 ratio was the only factor associated with the likelihood of achieving normalized CD4+ T-cell count. Previous studies have demonstrated similar results [9, 10, 30, 31, 33, 42–48].

CD8+ T-cell counts increased during the first year, but remained persistently elevated up until year 6. However, after 6 years, it decreased continuously in subsequent years of follow up on ART. CD8+ T-cell count appears to play important role in mortality in patients on ART [33, 49] and need to be monitored for clinical assessment.

CD4/CD8 ratio was the only biomarker that showed continuous increase throughout the follow up period. Like the CD4+ T-cell trend, however, only 12.3% of the cohort has normalized ratio (≥ 1.0) and few patients (8.8%) were able to attain a median reference ratio value of HIV-negative Ethiopians [35]. Whereas the initial increase in the CD4/CD8 ratio between ART initiation and year 5 of follow-up might be attributed to the continuous increase in the CD4+ T-cell count as CD8+ T-cells remained persistently elevated. However, in the later phase of ratio increase (i.e. year 6 to 14) might be attributed to the continuous decrease in CD8+ T-cells as the CD4+ T-cell count in the same period plateaued. Previous report from HICs showed trends whereby the increase of CD4 counts and decrease of CD8 counts appeared to be similar [8, 11, 30]. Nonetheless, the trends to normalization time in the Ethiopian cohort appears to be much slower than those in HICs, similar to the cohort in Uganda [14]. In addition, the time to reach plateau was even shorter in the HICs when compared to our results.

Our data demonstrate that the measurement of both CD4+ and CD8+ T-cells, along with the assessment of the CD4/CD8 ratio are highly relevant in long-term follow-up and evaluation of patients with respect to immune recovery. Taken together, all T-cell biomarkers (i.e. CD4+ T-cell count, CD8+ T-cell count and CD4/CD8 ratio) failed to normalize to the levels of healthy individuals despite long-term virologically suppressive ART.

The mechanisms related to incomplete immune recovery in patients on ART despite suppressive ART remains to be elucidated. Several studies have demonstrated that chronic immune activation [10, 20, 21, 30], older age [24], male sex [50, 51], background genetic differences [22] might play significant role.

Our study has several strengths. First, the cohort included a long-term of follow-up of HIV-1 patients on sustained suppressive ART for more than a decade. Second, data on immune recovery biomarkers was availability both at initiation of ART and also time-dependent changes in CD4+ T-cell, CD8+ T-cell counts and CD4/CD8 ratio were obtained. Third, to the best of our knowledge, our study included long-term analysis of CD4/CD8 ratio in patients under suppressive ART in the setting of SSA. Nonetheless, limitations related to our study include that our findings cannot be generalized to the entire population as the data is generated from only one study site. Furthermore, the retrospective design of the study has limitations in respect to completeness of available data and the small sample size is a potential weakness of the study. In particular relevant data that are potentially confounders, such as parasitic infections and malnutrition were not available or incomplete. Finally, the number of patients during follow-up of such long duration is compounded by patient attrition. Thus, interpretation of our data after 10 years where a substantial number of patient followed is decreased showing unstable trends.

### Conclusion

Our data demonstrate that the measurement of both CD4+ and CD8+ T-cells, along with the assessment of the CD4/CD8 ratio is highly relevant in long-term follow-up and evaluation of patients with respect to immune recovery. In addition, our findings show that the majority (76%) of the patients started ART with a CD4+ T-cell lower than 200 cells, and all the T-cell biomarkers (CD4+ T-cell count, CD8+ T-cell count and CD4/CD8 ratio) did not normalize to the levels of healthy individuals despite long-term virologically suppressive ART. The results emphasize the relevance of initiation of ART at the early stage of HIV-1 infection [51–53].

Whether current “treat-all” ART guidelines [54] will result in complete immunological recovery in the setting of SSA should be investigated. In addition, continuous increase in CD4/CD8 ratio while CD4+ T-cell count plateau after 5 years of ART initiation in our study suggests that monitoring ratio levels may serve as a better biomarker risk for disease progression monitoring among patients on long-term ART.

## Supporting information

S1 Fig Proportion of patients with different CD4/CD8 ratio cut-offs. (TIF)

## Abbreviations

ART: antiretroviral treatment
HICs: high-income countries
HIV: human immunodeficiency virus
IQR: interquartile range
LICs: low-income countries
SSA: Sub-Saharan Africa
