# Supplementary figures and images for: Immune recovery in HIV-1 infected patients with sustained viral suppression under long-term antiretroviral therapy in Ethiopia
Source: PLoS One. 2020 Oct 22;15(10):e0240880. doi: 10.1371/journal.pone.0240880 (PMC7580989; doi:10.1371/journal.pone.0240880)

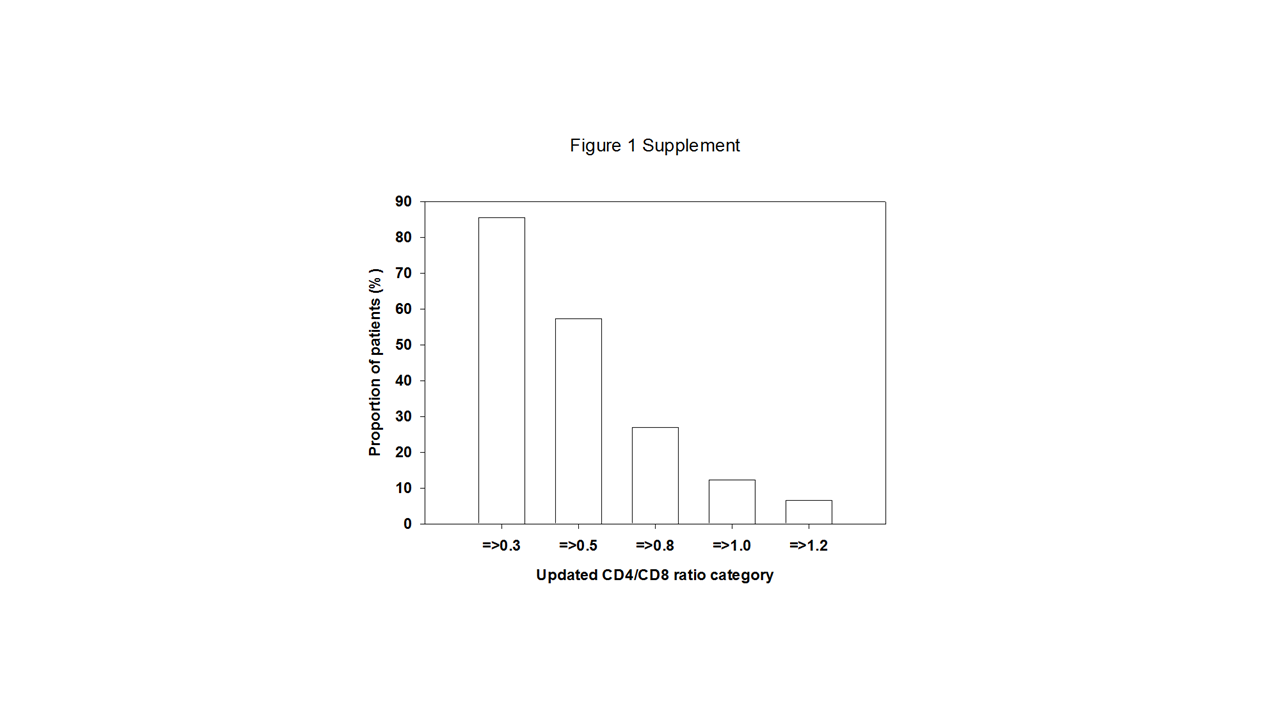

Supplement: S1 Fig — (TIF) [file pone.0240880.s001.tif]
